# Judicious Toggling of mTOR Activity to Combat Insulin Resistance and Cancer: Current Evidence and Perspectives

**DOI:** 10.3389/fphar.2016.00395

**Published:** 2016-10-25

**Authors:** Pei Shi Ong, Louis Z. Wang, Xiaoyun Dai, Sheng Hsuan Tseng, Shang Jun Loo, Gautam Sethi

**Affiliations:** ^1^Department of Pharmacy, Faculty of Science, National University of SingaporeSingapore, Singapore; ^2^Department of Pharmacology, Yong Loo Lin School of Medicine, National University of SingaporeSingapore, Singapore

**Keywords:** mechanistic target of rapamycin, insulin resistance, cancer, cancer stem cell, chemoresistance, rapamycin, mechanistic target of rapamycin inhibitors

## Abstract

The mechanistic target of rapamycin (mTOR), via its two distinct multiprotein complexes, mTORC1, and mTORC2, plays a central role in the regulation of cellular growth, metabolism, and migration. A dysregulation of the mTOR pathway has in turn been implicated in several pathological conditions including insulin resistance and cancer. Overactivation of mTORC1 and disruption of mTORC2 function have been reported to induce insulin resistance. On the other hand, aberrant mTORC1 and mTORC2 signaling via either genetic alterations or increased expression of proteins regulating mTOR and its downstream targets have contributed to cancer development. These underlined the attractiveness of mTOR as a therapeutic target to overcome both insulin resistance and cancer. This review summarizes the evidence supporting the notion of intermittent, low dose rapamycin for treating insulin resistance. It further highlights recent data on the continuous use of high dose rapamycin analogs and related second generation mTOR inhibitors for cancer eradication, for overcoming chemoresistance and for tumor stem cell suppression. Within these contexts, the potential challenges associated with the use of mTOR inhibitors are also discussed.

## Introduction

**Figure 1:**
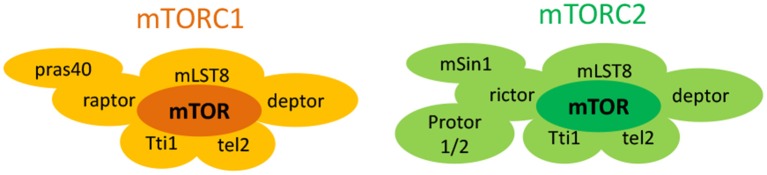
**Structure of mTORC1 and mTORC2 complexes**.

The mechanistic target of rapamycin (mTOR) is an atypical 289-KDa cytoplasmic serine/threonine protein kinase that belongs to the phosphoinositide 3-kinase (PI3K)-related kinase family of molecules (Laplante and Sabatini, 2012). It forms at least two structurally distinct multiprotein complexes, namely mTOR complex 1 (mTORC1) and mTOR complex 2 (mTORC2), with differing protein composition and subcellular localization (Figure 1; Laplante and Sabatini, 2012). mTORC1 comprises of six different companion proteins including the catalytic mTOR subunit, mammalian lethal with sec-13 (mLST8 or GβL; Kim et al., 2003), DEP domain mTOR-interacting protein (deptor; Peterson et al., 2009), Tti1/Tel2 complex (Kaizuka et al., 2010), regulatory-associated protein of mammalian target of rapamycin (raptor; Hara et al., 2002; Kim et al., 2002), and the proline rich AKT substrate 40 kDa (PRAS40; Sancak et al., 2007; Thedieck et al., 2007; Vander Haar et al., 2007; Wang et al., 2007). On the contrary, mTORC2 is formed from seven accessory units. Four of them (mTOR subunit, mLST8, DEPTOR, Tti1/Tel2 complex) are similar to mTORC1 while three others [rapamycin-insensitive companion of mTOR (rictor; Jacinto et al., 2004; Sarbassov et al., 2004), mammalian stress-activated map kinase-interacting protein 1 (mSin1; Frias et al., 2006; Jacinto et al., 2006), and protein observed with rictor 1 and 2 (protor1/2)] are unique to mTORC2 (Guertin et al., 2006; Pearce et al., 2007, 2011; Thedieck et al., 2007). These structural differences in turn affect their function, activation, and sensitivities to rapamycin, a prototype mTOR inhibiting fungicidal macrolide (Wullschleger et al., 2006).

**Figure 2:**
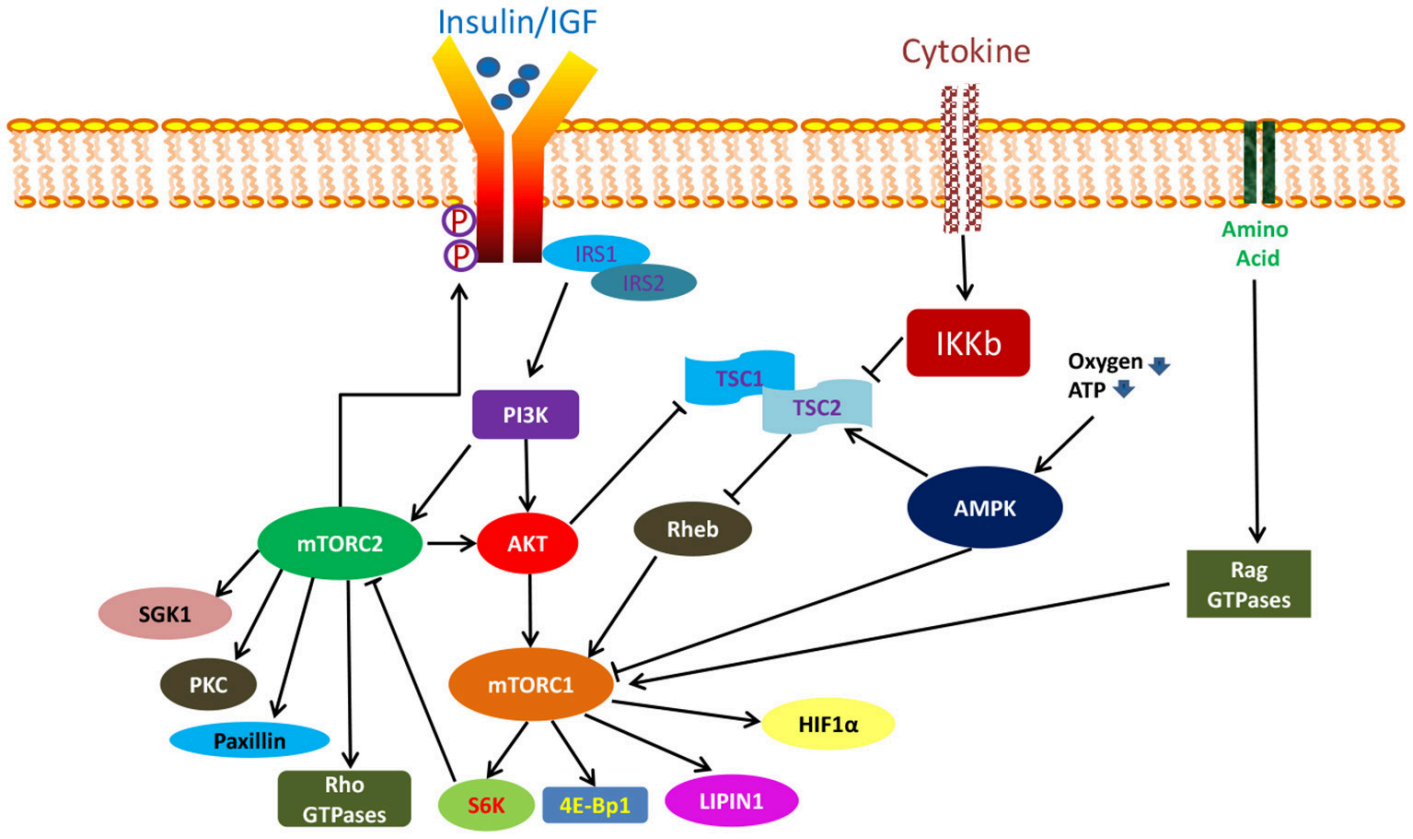
**mTORC1 and mTORC2 signaling**.

In general, mTORC1 and mTORC2 are functionally distinct kinases that activate separate but yet interconnected pathways with different upstream regulators and downstream targets (Figure 2) (Laplante and Sabatini, 2012). mTORC1 signaling has been more extensively studied and is better understood while much remains to be known about the mTORC2 pathway. Importantly, mTORC1 is particularly sensitive to acute rapamycin treatment while mTORC2, previously taught to be rapamycin insensitive, was subsequently found to be inhibited following chronic rapamycin exposure (Sarbassov et al., 2006).

mTORC1 plays a central role in integrating numerous upstream as well as downstream signals as a master controller. Its activity is regulated via a complex fashion which can be stimulated by insulin, growth factors, oxygen, high cellular energy level, amino acids, and cytokines (Feldman et al., 2009). Among them, insulin and growth factors stimulate the mTORC1 complex via the PI3K/AKT pathway (Laplante and Sabatini, 2012). Activated PI3K initiates a series of phosphorylation reactions which lead to the formation of phosphatidylinositol (3,4,5)-trisphosphate (PIP3). PIP3 in turn recruits serine threonine kinases such as protein kinase B (PKB also known as AKT) and 3-phosphoinositide-dependent protein kinase-1 (PDK1). The latter subsequently phosphorylate AKT at Th308, thereby enabling it to directly activate mTORC1 by promoting the dissociation of inhibitory PRAS40 protein from raptor (Inoki et al., 2002; Manning et al., 2002; Potter et al., 2002) or indirectly via the degradation of the tuberous sclerosis (TSC) 1/2 complex (Sancak et al., 2007; Thedieck et al., 2007; Vander Haar et al., 2007; Wang et al., 2007). TSC1/2 heterodimer is a negative regulator of mTORC1 which function by inactivating the mTORC1 activating small GTPAse, Ras homolog enriched in brain (RHEB) (Inoki et al., 2003a; Tee et al., 2003). On the other hand, oxygen and a high energy state activate mTORC1 via blocking the action of another mTORC1 negative regulator, adenosine monophosphate-activated kinase (AMPK). In its activated state, AMPK can directly block mTORC1 function, or works indirectly by promoting the mTORC1 inhibitory action of TSC2 (Inoki et al., 2003b; Gwinn et al., 2008). Amino acids, particularly leucine and arginine, activate mTORC1 through the Rag GTPases while cytokines act by IKB kinase-induced inactivation of TSC1/2 complex (Sancak et al., 2007; Kim et al., 2008). Regardless of activating source, activated mTORC1 phosphorylates its downstream effectors such as p70 S6 kinase 1 (S6K1) and eukaryotic initiation factor 4E binding protein 1 (4E-BP1; Ma and Blenis, 2009). It also acts on other molecules such as lipin and hypoxia-inducible factor (HIF1-aplha) (Hudson et al., 2002; Peterson et al., 2011). Through these mTORC1 substrates, mTORC1 promotes protein, nucleotide, and lipid biosynthesis in proliferating cells, thereby regulating their growth (Ben-Sahra et al., 2013; Robitaille et al., 2013). It also promotes mitochondrial biogenesis to positively drive cellular metabolism (Cunningham et al., 2007). Additionally, mTORC1 inhibits cellular catabolism via prevention of autophagy, thereby aiding in the maintenance of cellular homeostasis (Ganley et al., 2009; Hosokawa et al., 2009; Jung et al., 2009; Martina et al., 2012).

mTORC2 on the other hand is not responsive to nutrient simulation. It however responds to growth factors via a PI3K-mediated mechanism (Zinzalla et al., 2011). Upon activation, mTORC2 phosphorylate AKT at Ser473, serum- and glucocorticoid-induced protein kinase (SGK1), protein kinase C (PKC), and paxillin (Sarbassov et al., 2005; Guertin et al., 2006; García-Martínez and Alessi, 2008; Hagan et al., 2008; Gupta et al., 2013). It can also influence the activity of Rho GTPases (Jacinto et al., 2004). More recently, Yin and colleagues reported that mTORC2 can directly phosphorylate insulin receptor and insulin growth factor receptor at tyrosine residues to promote their activation (Yin et al., 2016). Therefore, through its effect on AKT, SGK1, insulin receptor and insulin-like growth factor receptor, mTORC2 influences cell survival, growth, and proliferation (Sarbassov et al., 2005; Guertin et al., 2006; García-Martínez and Alessi, 2008; Yin et al., 2016). In a similar manner, by regulating paxillin and Rho GTPases activities, mTORC2 also regulates the actin cytoskeleton and cell migration (Jacinto et al., 2004). Apart from these, mTORC2 activity can be regulated by mTORC1 where mTORC1-mediated activation of S6K1 has been shown to negatively influence mTORC2 function via phosphorylation of rictor and Sin 1 (Dibble et al., 2009; Julien et al., 2010; Liu P. et al., 2013).

Indeed, the mTOR complex plays an extremely vital role in many important cellular processes through the diverse actions of mTORC1 and mTORC2. A disturbance of the delicate balance within this pathway can lead to dire consequences such as the development of insulin resistance and cancer. Here, we discussed the present data supporting aberrant mTOR signaling as a crucial mechanism for triggering insulin resistance, tumorigenesis, chemoresistance, and cancer stem cells (CSCs) formation. We also emphasized on the need for prudent mTOR targeting to ameliorate insulin resistance. Additionally, an overview on the present status of the use of mTOR inhibitors for cancer and CSCs eradication will be provided.

## Role of mTOR in insulin resistance

mTOR plays a major role in nutrient sensing within the body. Under normal physiological state, increase in glucose and amino acid levels following a meal stimulates the secretion of insulin by the pancreatic β cells. Insulin begins its effect by binding to its cognate receptor, insulin receptor. Once activated, insulin receptor phosphorylate insulin receptor substrates 1 and 2 (IRS1 and IRS2) at its tyrosine residues, allowing them to associate with PI3K, thereby leading to the activation of downstream effectors such as AKT and protein kinase C (PKC; Laplante and Sabatini, 2012). AKT drives the metabolic action of insulin where it inactivates glycogen synthase 3 to mediate glycogen synthesis as well as the translocation of glucose transporter 4 (GLUT4) to the plasma membrane for glucose uptake into myocytes (Saltiel and Kahn, 2001; Pirola et al., 2004). Additionally, AKT activates mTORC1 (Raught et al., 2001) which in turn stimulates S6K1 to phosphorylate serine residues in IRS. This subsequently prevents the association of IRS with the insulin receptor (Paz et al., 1997; Gual et al., 2003). Such negative feedback mechanism thus desensitizes and protects the cell to further insulin simulation (Paz et al., 1997; Gual et al., 2003).

When mTORC1 is chronically activated following excessive glucose or branched chain amino acid consumption, the sustained simulation of downstream S6K1 increases IRS1 Ser307 phosphorylation. This decreases its activity and responsiveness to insulin, thereby rendering the cell insulin resistant (Morino et al., 2005; Tanti and Jager, 2009). Insulin resistance (IR) is typified by a decrease in the sensitivity of insulin responsive cells such as myocytes, adipocytes, hepatocytes, and pancreatic β-cells to normal circulating levels of insulin which can in turn be manifested as impair glucose tolerance and subsequently type 2 diabetes (Sesti, 2006; Bruce and Hanson, 2010). In mice lacking S6K1 in all tissues, resistance to diet induced insulin insensitivity and obesity were observed (Um et al., 2004). IRS1 protein expression is also seen to be markedly reduced in IR states (Saad et al., 1992; Goodyear et al., 1995). Furthermore, in mice lacking raptor in adipose tissue, an increased insulin sensitivity and resistance to diet-induced obesity were found (Polak et al., 2008). Conversely, constitutive activation of mTORC1/S6K1 through overexpression of RHEB or deletion of TSC1/2 led to a downregulation as well as enhanced proteosomal degradation of IRS1 and IRS2 (Shah et al., 2004). This reduces the signaling between insulin receptor and PI3K, thereby inducing IR (Hotamisligil et al., 1996; Liu et al., 2004; Shah et al., 2004; Tremblay et al., 2007; Blagosklonny, 2012). The sustained feedback via the mTORC1/S6K1 route has been confirmed by many others as a single most important mechanism for inducing IR (Tremblay and Marette, 2001; Harrington et al., 2004; Shah et al., 2004; Um et al., 2004; Nyman et al., 2014). Apart from this, activated mTORC1 can also directly promote IRS1/2 degradation to induce IR (Harrington et al., 2004; Briaud et al., 2005). More recently, another mechanism of negative feedback inhibition of insulin signaling has been reported. Here, mTORC1 has been shown to stabilize growth factor receptor-bound protein 10 (Grb10), a mTORC1 substrate, to facilitate its negative regulatory effects on insulin receptor, thereby rendering cells insensitive to insulin stimulation (Hsu et al., 2011; Yu et al., 2011). Through these three actions, an overactive mTORC1 in turn induces IR.

Apart from mTORC1, recent data suggest that mTORC2 can also promote IR either via its effect on IRS1 or by its ability to regulate AKT activation. When mTORC2 function is disrupted or silenced by SIN1 knockdown or small interfering RNA respectively, an elevated IRS1 level was observed (Kim et al., 2012). Further, analysis revealed that this elevation was the result of reduced IRS1 degradation mediated by ubiquitin ligase subunit Fbw8, a mTORC2 substrate, leading to the accumulation of inactive IRS1 in the cytosol (Kim et al., 2012). Despite the increase in IRS1 levels, IRS1 signaling in SIN1 deficient MEFs following insulin stimulation was reduced as evident by a reduced PIP3 formation (Kim et al., 2012). This data highlighted the important function of mTORC2 in influencing insulin sensitivity and resistance by coupling PI3K to IRS1 degradation (Kim et al., 2012). Another important role of mTORC2 in insulin action is mediated via AKT. A disruption of mTORC2 impairs insulin-induced activation of AKT, a mTORC2 substrate, causing a reduced insulin stimulated glucose uptake in both adipose tissue and muscles of rictor knockout mice (Cybulski et al., 2009; Kumar et al., 2010). It further resulted in increased gluconeogenesis as well as impaired glycolysis and lipogenesis in the liver of these animals (Hagiwara et al., 2012). Taken together, impaired AKT activation from mTORC2 disruption contributed to higher blood glucose levels and impaired glucose tolerance. All of these thus highlighted the crucial effect of mTOR in the development of IR.

## Role of mTOR in tumorigenesis and chemoresistance

As previously discussed, mTOR is a crucial regulator of cell growth and metabolism. This complex signaling pathway is tightly regulated in all normal cells. This is achieved through careful coordination of numerous molecules such as phosphatidylinositol-4,5-bisphosphate 3-kinase, catalytic subunit alpha (PIK3CA), AKT, phosphatase, and tensin homolog (PTEN), PDK1, TSC1/2, RHEB, and EIF4B. Dysregulation of some of them have been reported to induce pathological changes which have been implicated in malignant transformation of cells. Indeed, a deranged PI3K pathway can be found in almost all cancers. Up to 70% of ovarian cancer showed an activated PI3K/AKT/mTORC1 signaling (Li et al., 2014). This pathway is also activated in tumor tissues of patients with late stage gastric cancer compared to adjacent normal tissues (Tapia et al., 2014). At the molecular level, one of the factors contributing to increase activation of the PI3K/AKT/mTORC1 axis is the amplification or gain-of-function mutations of the PIK3CA gene. Such mutation is highly prevalent in cancers of the breast, colon, rectum, lung, ovary, cervix, and endometrium (Shayesteh et al., 1999; Ma et al., 2000; Samuels et al., 2004; Network, 2012a,b, 2013, 2014). Other gene aberrations such as overexpression of proto-oncogene, HER2, in breast cancer (Zhou et al., 2004), loss of function E17K mutation of AKT1 (Carpten et al., 2007; Do et al., 2008) and amplification of AKT2 in pancreatic cancer (Cheng et al., 1996; Ruggeri et al., 1998) that disrupt usual signaling along this pathway have been reported.

Aberrant mTORC1 activation can also be attributed to loss-of-function mutations of tumor suppressors including PTEN, liver kinase B1 (LKB1), and TSC1/2. In many cancers, the ability of PTEN, a negative regulator of AKT, to reduce the activity of mTORC1 via AKT is diminished when it is mutated, silenced, or deleted (Hollander et al., 2011). Likewise, a perturbed mTORC1 function resulting from LKB1 inactivation has been observed to lead to the formation of aggressive endometrial (Contreras et al., 2010) and non-small cell lung cancers (Mahoney et al., 2009). Inactivating non-sense, missense, frameshift, and splicing mutations in either TSC1 or TCS2 also remove their inhibitory effects on mTORC1, thereby leading to abnormal cell proliferation through an increased in S6K1 activity as well as an inhibition of the translation regulating effects of 4EBP1 (Consortium, 1993; Van Slegtenhorst et al., 1997). This is in turn manifested as TSC, a benign tumor disorder influencing many organs which can also results in metastatic lung cancer occasionally (Consortium, 1993; Van Slegtenhorst et al., 1997; Crino et al., 2007).

Besides functional changes in its upstream regulators that affect mTORC1 activity, a constitutively activated mTORC1 was originally reported by Sato and colleagues in intestinal adenocarcinoma and renal cell cancer (Sato et al., 2010). A more recent analysis of various human genome databases by Grabiner et al. revealed additional mutations in the C-terminal of the mTOR gene that can contribute to its hyperactivity (Grabiner et al., 2014). Furthermore, E4BP1/EIF4E, two components downstream of mTORC1 have also been associated with tumorigenesis. In an *in vitro* study by Dowling and co-workers, mouse embryonic fibroblasts that were depleted of E4BP1/EIF4E showed increased cell cycle progression and proliferation (Dowling et al., 2010). This result was further corroborated by the work of Hsieh et al. where E4BP1 was observed to mediate AKT-driven cancer initiation, growth and progression via the antiapoptotic Mcl-1 protein (Hsieh et al., 2010). These *in vitro* data was again supported by *in vivo* results where an overexpression of functionally active E4-BP1/EIF4E was found at the surgical margin of head and neck cancer. In these tumors, an increase AKT/mTOR signaling also resulted in higher tumor recurrence (Nathan et al., 2004). This observation was subsequently reinforced by other studies where a high E4BP1 level has been associated with high grade aggressive tumors with poorer prognosis in patients with breast, endometrial and ovarian cancers (Castellvi et al., 2006, 2009; Rojo et al., 2007; Darb-Esfahani et al., 2009).

Several other molecules have also been reported to activate mTORC1 and contribute to tumor formation. Absence of functional p53 has been shown to promote mTORC1 signaling and carcinogenesis (Feng et al., 2005). This could be attributed to a removal of negative regulation on mTORC1 since wild-type p53 protein has been shown to transactivate mTORC1 negative regulators such as TSC2 and AMPK (Feng et al., 2005). In a similar manner, loss of function mutations in GATOR1, a negative regulator of Rag GTPases which activate mTORC1 following amino acid stimulation, renders a hyperactive mTORC1 in tumor cells (Bar-Peled et al., 2013). On the contrary, overexpression of Rab1, a small GTPase that can activate mTORC1 following amino acid stimulation, promotes oncogenic growth, and progression in colorectal cancer (Thomas et al., 2014). Overall, all these data reinforce the involvement of mTORC1 in malignant transformation and tumor growth.

Increasingly, it is becoming clear that mTORC2 participates in tumorigenesis. In breast and prostate cancer cells with deliberate overexpression of mTORC2, a greater proliferation rate and metastatic profile were reported (Masri et al., 2007; Hietakangas and Cohen, 2008). This observation was reinforced by Lin and colleagues who found that mTORC2 is essential for heregulin mediated breast tumor formation via the receptor tyrosine kinase HER2/ErbB2 pathway (Lin et al., 2014). Two other groups of investigators provided further evidence supporting the role of a hyperactive mTORC2 in cancer through the manipulation of rictor. In astroglial cells, Bashir and coworkers showed that conditional overexpression of rictor led to the initiation and formation of malignant gliomas (Bashir et al., 2012). In another study by Guertin et al. using mouse prostate carcinoma induced by PTEN deletion, cancer development was in turn abrogated following rictor deletion (Guertin et al., 2009). This thereby implied that mTORC2 is essential for cellular hyperproliferation upon PTEN ablation induced increase in AKT activation. Overall, these *in vitro* observations were supported by *in vivo* findings where frequent overexpression of rictor was common in glioblastoma, breast and colorectal cancer (Sparks and Guertin, 2010).

Apart from the involvement of mTOR signaling in tumorigenesis, several reports also highlighted a role for mTOR in the development of resistance to anticancer therapies. For example, a constitutionally active AKT (Brognard et al., 2001; Clark et al., 2002; Vivanco and Sawyers, 2002) or chemotherapy-induced activation of AKT may render tumors resistant to anticancer therapies (Chakravarti et al., 2002; VanderWeele et al., 2004; VanderWeele and Rudin, 2005; Bozulic et al., 2008; Hurvitz et al., 2013). In breast cancer, a hyperactive AKT mediated by PTEN loss, PIK3CA, or AKT mutations resulted in resistance to anti-HER2 antibody, trastuzumab (Hurvitz et al., 2013). In glioblastoma treated with epidermal growth factor receptor inhibitors, drug resistance was mediated via amplification of AKT activity following a compensatory increase in insulin growth factor receptor signaling (Chakravarti et al., 2002). AKT have also been reported to mediate chemoresistance to microtubule inhibitors including vincristine and paclitaxel as well as topoisomerase II inhibitor such as doxorubicin (VanderWeele et al., 2004; VanderWeele and Rudin, 2005; Bozulic et al., 2008). The drug resistance propagated through AKT in turn have been shown to be dependent on mTOR signaling (VanderWeele et al., 2004; Wendel et al., 2004; VanderWeele and Rudin, 2005). It was further demonstrated to be specifically mediated through mTORC1 via increased translation of anti-apoptotic Mcl-1 protein (Mills et al., 2008). Besides mediating drug resistance through Mcl-1, chemoresistance can also be mediated through S6K1 (Yamnik et al., 2009). In estrogen receptor positive breast cancer, long term treatment with antiestrogen therapy resulted in up-regulation of mTOR signaling via the PI3K/AKT axis (Yue et al., 2003). The resultant increase in activated S6K1 can directly activate the estrogen receptor (ER) to increase transcription of ER-responsive genes independently of estrogen signaling, thereby rendering tumor cells unresponsive to endocrine therapy (Yamnik et al., 2009).

## Role of mTOR in cancer stem cell regulation

While the role of the PI3K-Akt-mTOR axis in cancer pathogenesis is well-established, its involvement in the regulation of CSCs has only become more evident in the last decade. CSCs represent a subpopulation of rare cancer cells that possess the inherent characteristics of normal stem cells. They can be derived from normal stem cells, lineage committed progenitor cells or non CSC tumor cells via the activation of numerous pathways of stemness, both known and unknown (Odoux et al., 2008; Chaffer et al., 2011; Gupta et al., 2011; Iliopoulos et al., 2011). Regardless of their source of origin, CSCs have the ability to self-renew, to undergo asymmetric cell division and to differentiate into the mass of heterogeneous, mature tumor cells. When transplanted into non-obese diabetic/severe immune deficient (NOD-SCID) mice, CSCs generate tumors resembling the histopathological features of the parental cancer (Singh et al., 2003). The first CSCs were identified in acute myeloid leukemia (AML; Lapidot et al., 1994; Bonnet and Dick, 1997). In solid tumors, CSCs have been found in numerous tumors including breast (Al-Hajj et al., 2003), colon (Ricci-Vitiani et al., 2007), prostate (Eaton et al., 2010), pancreas (Li et al., 2007), liver (Ma et al., 2007), glioblastoma (Galli et al., 2004; Singh et al., 2004; Stupp and Hegi, 2007), medulloblastoma (Hemmati et al., 2003; Singh et al., 2003; Galli et al., 2004), and lung cancers (Tirino et al., 2009). Evidence of the importance of mTOR in CSCs was initially derived from studies using genetically modified murine models. When the negative mTOR regulator, PTEN, was deleted in murine hematopoietic stem cells (HSCs), PTEN-mutant bone marrow showed rapid depletion of normal HSCs followed by an increased presence of leukemia initiating stem cells (LSCs or CSCs in leukemia) which subsequently resulted in leukemogenesis (Yilmaz et al., 2006; Zhang et al., 2006; Guo et al., 2008; Lee et al., 2010). This was abrogated using rapamycin treatment which resulted in ablation of LSCs together with restoration of normal HSCs, suggested that activation of mTORC1 was responsible for causing leukemia development while its normalization maintains HSC function (Yilmaz et al., 2006). Further, investigation with raptor or rictor deletion in PTEN deficient mice also reduced the severity of the cancer and extended their survival, thereby supporting the role of both mTORC1 and mTORC2 in murine LSCs survival (Kalaitzidis et al., 2012; Magee et al., 2012). In another murine model with increased mTOR signaling where HSCs were transduced with a constitutively active AKT, T-lymphoma developed in 65% of animals (Kharas et al., 2010). Subsequent treatment with rapamycin prolonged the survival of these mice, again reinforcing the importance of mTORC1 in influencing LSCs propagation (Kharas et al., 2010). Likewise, in primary human AML, Xu and colleagues showed that engraftment of AML stem cells in NOD/SCID mice were dramatically reduced in the presence of both rapamycin and etoposide compared to etoposide treatment alone, hence confirming that mTOR regulate AML stem cell survival following rapamycin treatment (Xu et al., 2005).

For solid cancers such as glioblastoma, prostate, lung and breast cancer, PTEN inactivation again conferred tumor cells with a CSC-like behavior. In PTEN deleted prostate stem and progenitor cell populations, cell expansion was initially observed which was followed by the initiation of prostate cancer (Wang et al., 2006). This data was further confirmed by Dubrovska and co-workers where PTEN knockdown in prostate CSC-like populations led to an increase in PI3K-mTOR activity as well as clonogenic and tumorigenic capacities of these cells (Dubrovska et al., 2009). Similarly, in breast CSCs, colony formation and tumor initiation abilities were mediated in PTEN deficient cells through activation of mTOR and downstream STAT3 signaling (Zhou et al., 2007). In non-small cell lung cancer cells, aberrant PI3K/AKT/mTOR activity resulted in increase in chemokine CXC4 level and ensuing STAT3 signaling that was responsible for maintenance of the stemness of CSCs in these tumors. This mTOR/CXCR4/STAT3 activity was augmented with PTEN mutation while introduction of wild type PTEN suppressed this signaling and CSC sphere formation, thus confirming the importance of mTOR in regulating CSCs survival (Jung et al., 2013). Subsequent observation further reinforced the crucial function of mTOR in CSCs survival where mTORC2 inhibition resulted in a decreased in CSCs marker, epithelial adhesion molecule and tumorigenicity of hepatocellular CSCs (Nishitani et al., 2013). In another recent study, Lamb and colleagues identified a 15-fold upregulation of an isoform of S6K (RPS6KB1) protein in CSCs-derived mammospheres from MCF-7 and T47D breast cancer cell lines (Lamb et al., 2015). The formation of these mammospheres were also reduced following treatment with rapamycin at nanomolar concentrations, suggesting that rapamycin mediated reduction in mTOR activity may reduce protein synthesis which can be used as a therapeutic strategy against CSCs (Lamb et al., 2015). Echoing the finding of Lamb and co-workers, Corominas-Faja et al. performed nuclear reprogramming of MCF-7 luminal-like breast cancer cells to *de novo* SOX2 overexpressing CSCs-like breast cancer cells (Corominas-Faja et al., 2013). The formation of breast cancer CSCs was accompanied with transcriptional repression of three mTOR suppressors (PKAA1, DDIT4/REDD1, and deptor) as well as upregulation of phosphorylated S6K protein levels, again signifying the activation of mTOR pathway in the acquisition of CSC-like cellular states (Corominas-Faja et al., 2013). As a whole, these observations underline the vital function of mTOR signaling in CSCs formation and regulation.

## Rapamycin and other agents for modulating mTOR activity to overcome insulin resistance

Chronic aberrant activation of mTOR promotes IR, thereby contributing to the development of metabolic diseases such as diabetes (Laplante and Sabatini, 2012). As a result, it has been suggested that inhibition of the mTOR pathway may potentially reduce IR and thus improve glucose homeostasis (Blagosklonny, 2012, 2013). However, the modulation of the mTOR pathway using mTOR inhibitors is unquestionably complicated. Continuous daily use of high dose mTOR inhibitors such as rapamycin in organ transplant (Miles et al., 1998; Kasiske et al., 2003) or rapalogs in cancer treatment have resulted in IR, glucose intolerance, increase gluconeogenesis, and diabetes-like syndrome in a subset of patients (Pallet and Legendre, 2013; Sivendran et al., 2014). This apparent contradiction has been postulated to be due to the U-shaped profile of mTOR activity where its deficiency or abundance is detrimental to the overall balance of cellular metabolism (Laplante and Sabatini, 2012). With this preamble, it has since been suggested that low dose intermittent administration of rapamycin may have beneficial effects in combating IR while minimizing the long term metabolic toxicities associated with its chronic continuous use (Blagosklonny, 2012, 2013).

Rapamycin, also known commercially as Sirolimus, is a macrocyclic lactone isolated from the soil bacterium Streptomyces hygriscopicus by Ayerst Pharmaceuticals (Table 1; Douros and Suffness, 1981). It is an allosteric inhibitor of mTOR that acts by first binding to 12 kDa FK506 binding protein (FKBP12), an accessory protein, which in turn binds to the FKBP-rapamycin-binding domain of mTOR, thereby forming a ternary complex with mTOR (Kunz and Hall, 1993; Chen et al., 1995; Choi et al., 1996). Once formed, the rapamycin-FKBP12 complex prevents the action of mTOR as well as disrupts its downstream signaling to mediate its mTOR inhibitory actions (Kunz and Hall, 1993; Chen et al., 1995; Choi et al., 1996). While rapamycin is widely believed to be an mTORC1 inhibitor, recent studies confirmed that it can also inhibit mTORC2 albeit more weakly than mTORC1 after prolonged treatment (Sarbassov et al., 2006). It is believed that this mTORC2 inhibitory action, which perpetuated a decrease in IRS degradation, AKT activation and mTORC2-induced insulin receptor signaling, is partially responsible for rapamycin-induced hyperinsulinemia, IR and diabetes-like syndrome after its chronic usage (Deblon et al., 2012; Lamming et al., 2012; Martina et al., 2012). This has been confirmed in hepatic rictor knockout mice where mTORC2 deficiency resulted in loss of AKT Ser473 phosphorylation, systemic hyperglycaemia, and hyperinsulinemia (Hagiwara et al., 2012). Additionally, in patients receiving long term rapamycin, impaired IRS signaling and AKT activation have been observed (Di Paolo et al., 2006).

**Table 1:** **Rapamycin and approved rapalogs for cancer treatment**.

**Inhibitor name**	**Company**	**Stage of development**	**Approved indications**	**References**
Rapamycin (Sirolimus) 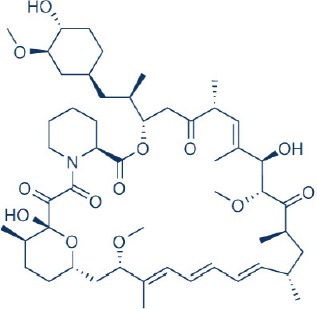	Wyeth/Pfizer	FDA Approved	Immunosuppressant for solid organ transplant, drug coated stent	Tsang et al., 2007; Benjamin et al., 2011; Wander et al., 2011
CCI-779 (Temsirolimus) 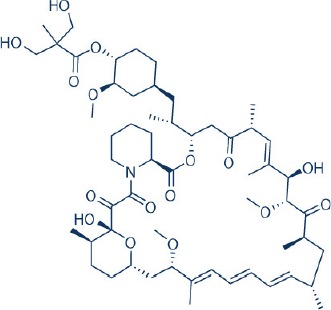	Novartis	FDA Approved	Renal cell carcinoma, relapsed or refractory mantle cell carcinoma	Hess et al., 2009; Benjamin et al., 2011; Wander et al., 2011
RAD001 (Everolimus) 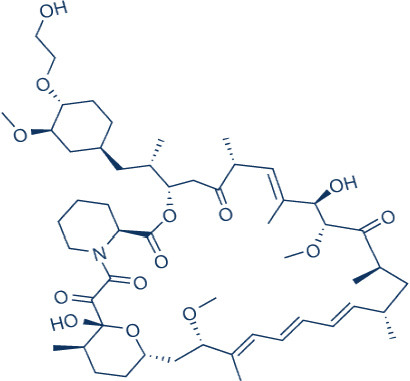	Wyeth/Pfizer	FDA Approved	Pancreatic neuroendocrine cancer, renal cancer, renal angiomyolipoma with TSC, subenpendymal giant cell astrocytoma with TSC, breast cancer	Motzer et al., 2008; Krueger et al., 2010; Benjamin et al., 2011; Wander et al., 2011; Yao et al., 2011; Bissler et al., 2013
AP23573 (Ridarolimus, formerly known as Deforolimus) 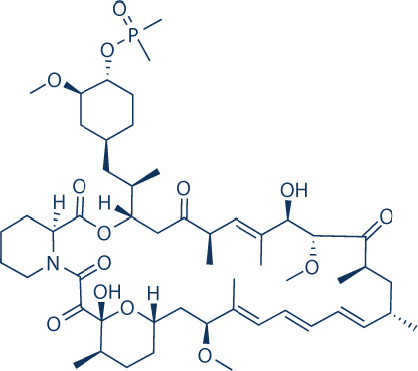	MERCK/ARIAD	Phase III; discontinued	–	Blay et al., 2012

Despite the IR and diabetes causing effects, its use has paradoxically been shown to reduce development and progression of diabetic complications as well as prolong longevity *in vivo* (Lloberas et al., 2006; Yang et al., 2007; Harrison et al., 2009; Mori et al., 2009; Reifsnyder et al., 2014; Xiao et al., 2014). The two latter observations have been linked to its mTORC1 deactivating action independently of its mTORC2 inhibitory effects (Gödel et al., 2011; Lamming et al., 2012), suggesting that it may be possible to achieve a balance between the beneficial and detrimental effects of mTOR inhibition associated with rapamycin use (Gödel et al., 2011). Acute rapamycin treatment inhibited mTORC1-mediated S6K1-IRS negative feedback loop and has been found to restore insulin signaling (Tremblay et al., 2005; Tzatsos and Kandror, 2006). Low dose intermittent rapamycin use may thus be useful in the management of IR as it avoids its mTORC2-mediated IR as well as other potential IR inducing effects such as inhibition of mitochondria biogenesis and decrease in YY1 gene expression following long term mTORC1 inhibition (Morino et al., 2006; Blagosklonny, 2012, 2013; Blättler et al., 2012)]. Indeed, this has been clearly observed in myotubes *in vitro* where acute treatment with rapamycin (< 1 h) resulted in increased insulin sensitivity through mTORC1 disruption while prolonged exposure (>24 h) render cells refractory to the actions of insulin via mTORC2 inhibition (Ye et al., 2012). In humans, insulin responsiveness and glucose update have also been improved following a single oral dose of rapamycin (Krebs et al., 2007). Further investigations are thereby required to determine a suitable, rational, intermittent low dose rapamycin dosing schedule to achieve the purported improvement in IR.

Apart from rapamycin, metformin, a derivative of biguanide from *Galega officinalis*, is a widely used antidiabetic agent known for its ability to reduce gluconeogenesis and IR. The latter is brought about by the ability of metformin to increase hepatic insulin sensitivity and glucose utilization by skeletal muscle (Pryor and Cabreiro, 2015). The initial molecular mechanism responsible for its IR lowering action was found to be due to AMPK activation via inhibition of complex 1 of the mitochondrial respiratory chain (Hardie, 2007). This is in turn the result of an indirect inhibition of mTORC1 signaling (Zhou et al., 2001; Shaw et al., 2005). More recently, Kalender and colleagues showed that metformin induced glucose uptake by myocytes occurred via direct inhibition of mTORC1 which occurred independently of AMPK (Kalender et al., 2010). Other agents including thiazolidinediones (He et al., 2006) and resveratrol (Baur et al., 2006) can also indirectly inhibit mTOR activity via activation of AMPK to reduce IR. Taken together, these findings highlighted that appropriate modulation of mTOR activity using suitable agents may be useful for overcoming IR.

## mTOR inhibitors for modulating mTOR activity to combat cancer and chemoresistance

Tumor growth driven by hyperactivation of mTOR is widely prevalent in numerous cancer types. As such, this kinase has become a vital therapeutic target. Numerous mTOR inhibitors have since been developed as anticancer therapies for modulating mTOR activity in tumors. The first generation of mTOR inhibitor is prototyped based on its founding member rapamycin. This is in partially attributed to the excellent antiproliferative activity of rapamycin against a wide variety of tumors including mammary, colon, and brain cancers *in vitro* and *in vivo* (Douros and Suffness, 1981) during preliminary testing by the National Cancer Institute in the United States. In the preclinical studies *in vitro* using cancer cell lines, rapamycin primarily acted as a cytostatic agent to arrest cell growth. Among some of the cell lines tested, rapamycin treatment also leads to apoptosis induction and showed antiangiogenesis properties (Muthukkumar et al., 1995; Shi et al., 1995; Ahn et al., 1997; Hosoi et al., 1999; Guba et al., 2002; Huang et al., 2004). Its co-treatment further enabled chemosensitization of the anticancer effects of other chemotherapeutics (Shi et al., 1995). All these attractive antineoplastic actions led to the initiation of numerous clinical studies investigating its anticancer effects (Zhang et al., 2011). Results from these clinical trials have however been disappointing where it showed clinical efficacy only in limited types of tumors accompanied by a highly variable therapeutic response (Zhang et al., 2011). This less than optimal outcome is in part attributed to the poor water solubility and stability of rapamycin.

In order to overcome the inferior pharmacokinetic properties of rapamycin and to further explore the therapeutic utility of mTOR inhibition to combat cancer, numerous analogs of rapamycin were subsequently developed based on the structure of rapamycin as a molecular scaffold. These analogs are commonly known as rapalogs which act via the same mechanism of action as rapamycin by predominately inhibiting mTORC1. They also showed superior pharmacokinetic properties in comparison with rapamycin and have reduced immunosuppressive effects (Ballou and Lin, 2008; Rizzieri et al., 2008). The three major rapalogs that have been developed to date for cancer treatment are shown in Table 1.

The first rapalog that was developed is CC1779, also known as temsirolimus. It is a 42-[2,2-bis (hydroxymethyl)]-propionic ester of rapamycin that demonstrated better water solubility and stability 2004. It has been formulated for both intravenous use and oral administration. While temsirolimus is converted to rapamycin as the major metabolite *in vivo*, temsirolimus itself has also been shown to possess antitumor effects, thereby conferring it with a dual mTOR inhibitory action (Hutson et al., 2008). In pre-clinical studies, temsirolimus delayed tumor growth of a diverse range of cancer types including glioblastoma, medulloblastoma, breast carcinoma, and renal cell carcinoma (2004; Martin et al., 2012). In particular, temsirolimus showed increased sensitivity to tumors harboring PTEN deletion, normalized their S6K1 activity and reduced these tumor growth (Neshat et al., 2001; Podsypanina et al., 2001). In early therapeutic trials, response for temsirolimus was the most promising in renal cell carcinoma (Atkins et al., 2004). Here, it was observed that patients with intermediate and poor risk responded better to temsirolimus therapy. This thereby led to the initiation of a subsequent phase III study of temsirolimus alone vs. interferon-alpha (IFN-alpha) alone vs. combination therapy of the two drugs for treatment of renal cell carcinoma patient with poor risk (Hudes et al., 2007). The superior overall survival of temsirolimus treated group vs. the IFN –alpha treated arm in this study in turn facilitated its approval as a first line therapy for advanced renal cell carcinoma by the United States Food and Drug administration (FDA) and European Medicines Agency (EMEA) in 2007. Apart renal cell carcinoma, temsirolimus has also shown good activity against relapsed or refractory mantle cell lymphoma and has since been approved by EMEA for this indication (Hess et al., 2009). Despite the clinical efficacy of temsirolimus in these two cancers, its effects in other solid tumors such as neuroendocrine tumor, breast, and lung cancers as a single agent are however modest (Duran et al., 2006; Pandya et al., 2007; Wolff et al., 2013).

RAD001 (everolimus) or 42-O-(2-hydroxyethyl)-rapamycin is another rapalog that has been developed. It is more polar and has improved oral bioavailability compared to rapamycin (Fasolo and Sessa, 2008). *In vitro* study of everolimus in 24 tumor cell lines demonstrated that everolimus exhibited potent antiproliferative activity with a median IC50 value of 8.8 nM (Fasolo and Sessa, 2008). In particular, everolimus exhibited increased tumor kill against cancer cell lines harboring high levels of AKT (Boulay et al., 2003). Likewise, in syngeneic and orthotropic animal models, everolimus showed significant tumor growth suppression and antiangiogenic activity (Schuler et al., 1997; O'Reilly et al., 2002; Beuvink et al., 2005; Mabuchi et al., 2007). This data was echoed in phase II clinical studies where everolimus achieved a good response rate of 47 and 30% in Hodgkin lymphoma and non-Hodgkin's lymphoma respectively (Johnston et al., 2010; Witzig et al., 2011). Other tumors where everolimus showed promising activity in early phase studies include anaplastic large cell lymphomas, pancreatic neuroendocrine tumor, metastatic renal cell carcinoma and ovarian cancer (Fasolo and Sessa, 2008). Subsequent phase III trials of everolimus as a single agent showed significant improvement in progression free survival (PFS) or response rates in advanced pancreatic neuroendocrine tumors, advanced renal cell carcinoma, renal angiomyolipoma with TSC and subependymal giant cell astrocytoma with TSC (Motzer et al., 2008; Krueger et al., 2010; Yao et al., 2011; Bissler et al., 2013). Everolimus has since been approved by FDA for these tumors. More recently, similar favorable outcome has also been reported for everolimus monotherapy in neuroendocrine tumors of the lung and gastrointestinal tract (Yao et al., 2016). Despite its encouraging data in these tumors, for other cancers where everolimus has been investigated as a monotherapy, poor efficacy has been observed (Tarhini et al., 2010; Ohtsu et al., 2013; Llovet and Hernandez-Gea, 2014; Mego et al., 2016).

Following the development of temsirolimus and everolimus, a third rapalog, AP23573 or dimethylphosphinic acid rapamycin-40-O-yl ester (ridaforolimus, formerly known as deforolimus) has also been developed. Unlike temsirolimus, it is not a rapamycin prodrug and has greater stability compared to the other rapalogs (Metcalf et al., 2004). Ridaforolimus showed antiproliferative actions against glioblastoma, prostate, breast, pancreas, lung and colon cancer cell lines as well as in mouse xenograft models (Clackson et al., 2003). Subsequent, early clinical trials of this rapalog verified its antitumor activity in endometrial cancer, malignant glioma and sarcoma (Mita et al., 2008). The encouraging results of ridaforolimus in phase II sarcoma studies also led to the phase III SUCCEED clinical trial of ridaforolimus in soft tissue and bone sarcoma. In this study, single agent ridaforolimus resulted in improved progression-free survival (PFS) compared to placebo (Blay et al., 2012). Its development as an anticancer agent was however halted after rejection of its new drug application by FDA in 2012.

Overall, it can be observed that the effects of rapalogs are limited to a subset of solid tumors. The reasons for this moderate activity may be due to their failure to inhibit mTORC2, their incomplete inhibitory effects on mTORC1 and their induction of feedback activation that resulted in increased upstream tyrosine kinase signaling, AKT upregulation as well as enhanced activity of other survival pathways (Sun et al., 2005; O'Reilly et al., 2006; Carracedo et al., 2008; Wang et al., 2008; Zhang et al., 2011). As a consequence, it has been postulated that the use of dual specific molecules that can target both mTORC1 and mTORC2 to prevent mTORC-2 driven AKT activation may provide better efficacy at modulating mTOR activity in tumors.

A new generation of mTOR inhibitors, also known as mTOR kinase inhibitors, that acts via binding to the ATP-binding site of mTOR kinase competitively has since emerged (Benjamin et al., 2011; Wander et al., 2011). These inhibitors act by inhibiting the catalytic activity of both mTORC1 and mTORC2 (Benjamin et al., 2011; Wander et al., 2011). They can be classified into two groups namely, selective mTORC1/mTORC2 inhibitors and dual specificity PI3K and mTOR kinase inhibitors. The latter acts on PI3K in addition to mTORC1 and mTORC2. To date, more than 10 selective mTORC1/mTORC2 inhibitors have been identified (Table 2; Gokhale et al., 2008; García-Martínez et al., 2009; Yu et al., 2009; Chresta et al., 2010; Liu et al., 2010, 2012; Liu Q. et al., 2013; Bhagwat et al., 2011; Hsieh et al., 2012; Hart et al., 2013; Pike et al., 2013; Takeuchi et al., 2013; Mortensen et al., 2015a,b,c).

**Table 2:** **Investigational selective mTORC1/mTORC2 inhibitors**.

**Inhibitor name**	**Company**	**Stage of development**	**Approved indications**	**References**
PP242 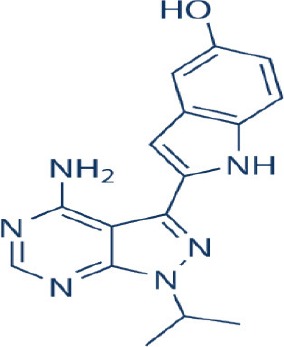	–	Preclinical; Discontinued	Leukemia, multiple myeloma, myeloproliferative neoplasms, breast, cancer, endometrial, esophageal squamous cell, gastric, hepatocellular, ovarian cancer, glioblastoma, medulloblastoma, osteosarcoma, paragangliomas, pheochromocytomas	Sun, 2013b
INK-128/MLN0128 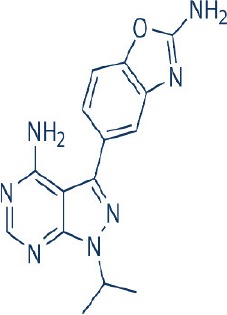	Intellikine	Phase I, II	Acute lymphoblastic leukemia, multiple myeloma, non-Hodgkin's lymphoma, breast, colon, hepatocellular, lung, pancreatic, prostate, renal, thyroid cancer, bone and soft tissue sarcoma, euroblastoma, glioblastoma, osteosarcoma, Merkel cell carcinoma	Benjamin et al., 2011; Wander et al., 2011; Zhang et al., 2011; Ghobrial et al., 2012; Hsieh et al., 2012; Sun, 2013b
AZD8055 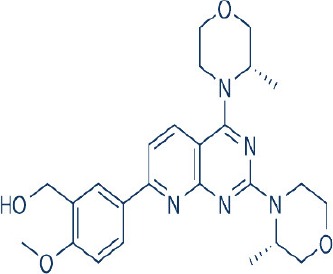	Astra Zeneca	Phase I, II	Leukemia, lymphomas, multiple myeloma, breast, cervical, colon, endometrial, gallbladder, head and neck, hepatocellular, laryngeal, lung, ovarian, prostate, renal, pancreatic cancer, gliomas, pheochromocytoma rhabdomyosarcoma, uterine serous carcinoma	Chresta et al., 2010; Benjamin et al., 2011; Wander et al., 2011; Zhang et al., 2011; Naing et al., 2012; Asahina et al., 2013; Sun, 2013b
AZD2014 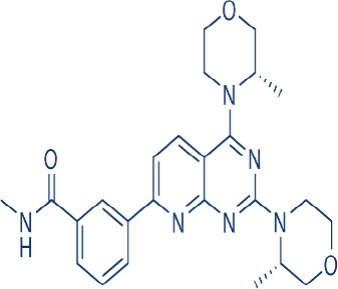	Astra Zeneca	Phase I, II	Leukemia, lymphoma, breast, colon, gastric, hepatocellular, lung, prostate cancer, female reproductive system neoplasm	Benjamin et al., 2011; Zhang et al., 2011; Banerji et al., 2012; Sun, 2013b; Basu et al., 2015; Liao et al., 2015; Powles et al., 2016
OSI027 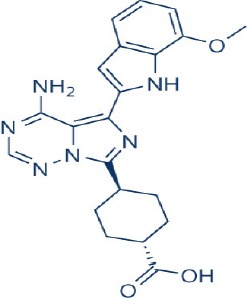	OSI	Phase I; Discontinued	Leukemia, lymphoma, bladder, breast, colon, head and neck, hepatocellular, lung, ovarian, pancreatic, prostate cancer	Tan et al., 2010; Bhagwat et al., 2011; Benjamin et al., 2011; Wander et al., 2011; Zhang et al., 2011; Sun, 2013b
Torin 1 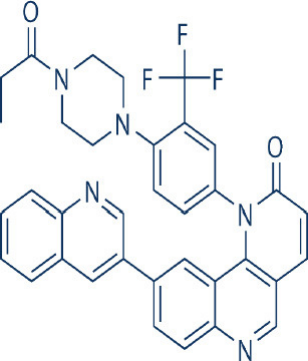	–	Preclinical	Colon, pituitary, neuroendocrine cancer, glioblastoma	Liu et al., 2010; Sun, 2013b
Torin 2 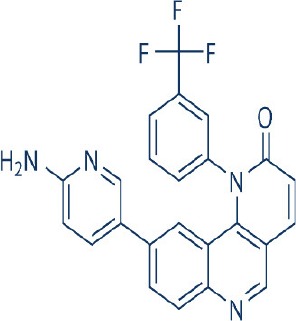	–	Preclinical	Leukemia, lung, hepatocellular, ovarian, thyroid cancer, papillary thyroid carcinoma, glioblastoma	Liu et al., 2012; Sun, 2013b
WAY600 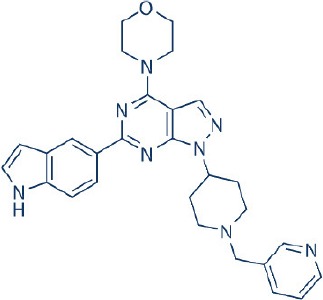	Wyeth/Pfizer	Preclinical	Breast, colon, prostate, renal cancer, glioblastoma	Sun, 2013b
WYE687 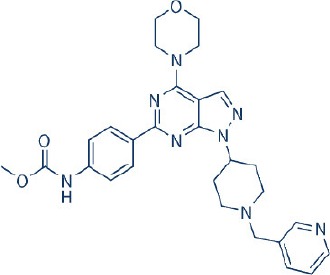	Wyeth/Pfizer	Preclinical	Breast, colon, prostate, renal, glioblastoma	Sun, 2013b
WYE354 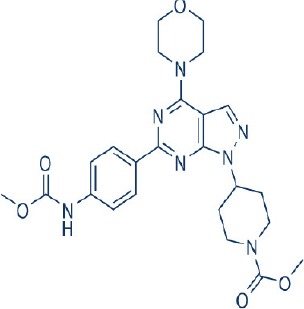	Wyeth/Pfizer	Preclinical	Breast, colon, gallbladder, prostate, renal cancer, glioblastoma	Yu et al., 2009; Sun, 2013b
Ku0063794 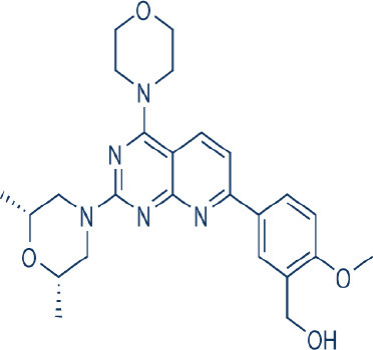	Kudos	Preclinical	Bladder, hepatocellular, lung cancer	García-Martínez et al., 2009; Sun, 2013b
CC223 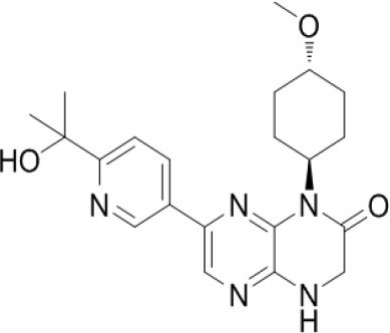	Celgene Corporation	Phase I	Leukemia, multiple myeloma, non-Hodgkin's lymphoma, breast, colon, hepatocellular, lung, prostate, renal cancer, glioma	Benjamin et al., 2011; Schatz, 2011; Shih et al., 2012; Goy et al., 2013; Varga et al., 2013; Bendell et al., 2015a; Mortensen et al., 2015a,c
C115 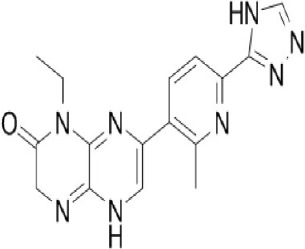	Celgene Corporation	Phase I, II	Leukemia, head and neck, prostate cancer, osteosarcoma, glioblastoma	Schatz, 2011; Mortensen et al., 2015b
XL388 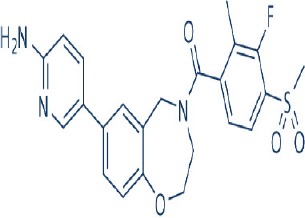	Exelixis Inc.	Preclinical	Breast cancer	Takeuchi et al., 2013
OXA01 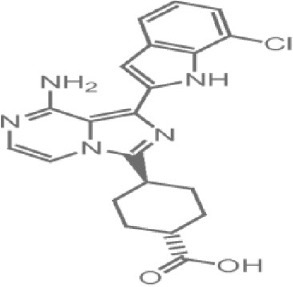	OSI	Preclinical	Colon, pancreatic cancer	Gokhale et al., 2008

Among them, PP242, Torin 1, Torin 2, WAY600, WYE-687, WYE-354, and Ku0063794 are still in the preclinical development stage while early phase clinical studies have been initiated for MLN0128, AZD8055, AZD2014, OSI-027, CC-223, and CC-115 (Table X) (Gokhale et al., 2008; García-Martínez et al., 2009; Yu et al., 2009; Liu et al., 2010, 2012; Liu Q. et al., 2013; Tan et al., 2010; Bhagwat et al., 2011; Schatz, 2011; Banerji et al., 2012; Ghobrial et al., 2012; Naing et al., 2012; Shih et al., 2012; Asahina et al., 2013; Goy et al., 2013; Pike et al., 2013; Takeuchi et al., 2013; Varga et al., 2013; Basu et al., 2015; Mortensen et al., 2015a,b,c). More importantly, majority of members of this class of mTOR inhibitors showed high potency against mTOR with half maximal inhibitory concentration against mTORC1 and mTOCRC2 in the low nanomolar concentrations (Feldman et al., 2009; García-Martínez et al., 2009; Chresta et al., 2010; Hsieh et al., 2012; Liu et al., 2012). They also exhibited a high degree of selectivity against mTOR kinase in comparison with other kinases such as PI3K that share similar structural homology at their kinase domains (Feldman et al., 2009; García-Martínez et al., 2009; Chresta et al., 2010; Hsieh et al., 2012; Liu et al., 2012). In preclinical studies, selective mTORC1/mTORC2 inhibitors such as PP242, WYE354, AZD8055, and Torin 1 demonstrated superior efficacies at inhibiting mTORC1-dependent phosphorylation of S6K1, 4EBP1 and mTOCR2-dependent activation of AKT at Ser473 compared with rapamycin (Feldman et al., 2009; Thoreen et al., 2009). This was in turn translated into greater reduction of cell proliferation and protein translation in addition to increased G1 cell cycle arrest or/and apoptosis induction compared to rapamycin (Feldman et al., 2009; Thoreen et al., 2009). Beside these, some members of this group such as AZD8055 and AZD2014 are capable of propagating cell kill through autophagy and had shown antimetastatic properties via inhibition of AKT mediated p27 phosphorylation (García-Martínez et al., 2009; Wander et al., 2011; Liao et al., 2015). All in all, these compounds exhibited anticancer effects against a wide range of cancer cell types including acute leukemia, multiple myeloma, breast, colon, lung, prostate, and renal cancer in both cancer lines and mouse xenograft models (Sun, 2013b). Their superior antitumor activity in preclinical studies in comparison with rapalogs has led to further clinical testing of some of these compounds.

For the agents in the clinical phases of development, phase I dose finding data is available for MLN0128, AZD8055, AZD2014, and CC-233 (Tan et al., 2010; Banerji et al., 2012; Ghobrial et al., 2012; Naing et al., 2012; Shih et al., 2012; Asahina et al., 2013; Goy et al., 2013; Varga et al., 2013; Basu et al., 2015; Bendell et al., 2015a). In a phase I dose escalation study of MLN0128 in patients with relapsed or refractory multiple myeloma, non-Hodgkin's lymphoma or waldenstrom macroglobulinemia, oral MLN0128 was generally well-tolerated, with a limited incidence of hyperglycemia development (Ghobrial et al., 2012). Among 37 patients enrolled, 27 showed confirmed responses with greater antitumor effects observed for patients given more frequent dosing schedules (Ghobrial et al., 2012). For AZD8055, phase I studies identified the maximum tolerated dose (MTD) to be 90 mg twice daily in both Caucasian and Japanese patients with advanced solid tumors or lymphoma (Naing et al., 2012; Asahina et al., 2013). AZD8055 showed an acceptable toxicity profile in general with an exception of ~22% of patients displaying elevated transaminase levels (Naing et al., 2012). Transient decreased in p4EBP1 was also observed in 40% of patient receiving the MTD of AZD8055 (Naing et al., 2012). Although no complete or partial responses were observed, 18% of patients had stable disease for more than 4 months (Naing et al., 2012). In the first-in-human phase I trial of AZD2014 in solid tumors, a MTD of 50 mg twice daily was obtained with reduction in phosphorylation of S6K, 4EBP1, and pAKT observed in patient biopsies (Banerji et al., 2012; Basu et al., 2015). In this study, a RECIST partial response was seen in one patient with acinar pancreatic cancer (Banerji et al., 2012). For phase I study of OSI-027, initial evidence of pharmacological activity was observed in 26% of treated patients showing stable disease lasting more than 12 weeks (Tan et al., 2010). As with the other selective mTORC1/mTORC2 inhibitors, CC-233 was well-tolerated and demonstrated comparable toxicities with other members of the class (Shih et al., 2012; Goy et al., 2013; Varga et al., 2013; Bendell et al., 2015a). Currently, phase II studies were underway for MLN0128, AZD8055, and AZD2014 (Sun, 2013b; Powles et al., 2016). More recently, the results of a phase II trial of AZD2014 vs. everolimus in patient with VEGF-refractory metastatic clear cell renal cancer were reported (Powles et al., 2016). In this study, AZD2014 unexpectedly showed an inferior progression free survival (PFS) compared with everolimus (PFS for AZD2014 = 1.8 months vs. PFS for everolimus = 4.6 months, *p* = 0.01; Powles et al., 2016). At present, the exact reason for the poorer efficacy of AZD2014 is not clearly known. This disparity in results has however been attributed to the additional effects on the tumor microenvironment and anti-angiogenesis activity of everolimus compared with AZD2014 (Powles et al., 2016).

Apart from selective mTORC1/mTORC2 inhibitors, dual specificity PI3K and mTOR kinase inhibitors are another subgroup of second generation mTOR inhibitors under development. Unlike selective mTORC1/mTORC2 inhibitors, these compounds inhibit both mTOR kinases and PI3K at similar effective concentrations (Benjamin et al., 2011). They have the advantage over selective mTORC1/mTORC2 inhibitors in that they are capable of overcoming PI3K mediated survival signals that arise as a consequence of mTORC1 inhibition, thereby minimizing drug resistance associated with the use of the selective mTORC1/mTORC2 inhibitors (Feldman et al., 2009). At present, at least 10 dual PI3K-mTOR inhibitors are in various stages of preclinical or/and clinical testing (Table 3) (Maira et al., 2008; Park et al., 2008; Schnell et al., 2008; Serra et al., 2008; Brachmann et al., 2009; Cao et al., 2009; Guillard et al., 2009; Liu et al., 2009, 2015; Marone et al., 2009; McMillin et al., 2009; Cho et al., 2010; Dolly et al., 2010; Knight et al., 2010; Mallon et al., 2010, 2011; Wagner et al., 2011; Wallin et al., 2011; Yuan et al., 2011; Mahadevan et al., 2012; Markman et al., 2012; Wang F.-Z. et al., 2013; Britten et al., 2014; Hong et al., 2014; Kashiyama et al., 2014; Makker et al., 2014; Papadopoulos et al., 2014, 2015; Powles et al., 2014; Yokota et al., 2014; Yu et al., 2014; Salazar et al., 2015; Bendell et al., 2015b; Chen et al., 2016; Gravina et al., 2016; Munster et al., 2016; Seront et al., 2016; Thijssen et al., 2016).

**Table 3:** **Investigational dual PI3K/mTOR inhibitors**.

**Inhibitor Name**	**Company**	**Stage of development**	**Approved indications**	**References**
BEZ-235 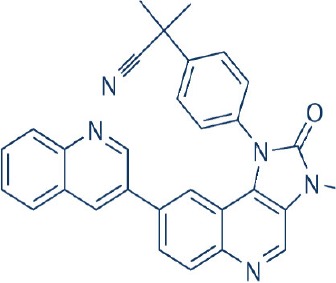	Novartis	Phase I, II; discontinued	Leukemia, lymphoma, multiple myeloma, adrenocortical, bladder, breast, colon, cholangiocarcinoma, endometrial, gastric, hepatocellular, lung, nasopharyngeal, ovarian, pancreatic, prostate, renal, thyroid cancer, gliomas, melanoma, non-functioning pituitary adenomas, osteosarcoma, rhabdomyosarcoma	Maira et al., 2008; Schnell et al., 2008; Serra et al., 2008; Brachmann et al., 2009; Cao et al., 2009; Liu et al., 2009; Marone et al., 2009; McMillin et al., 2009; Cho et al., 2010; Benjamin et al., 2011; Wander et al., 2011; Zhang et al., 2011; Hong et al., 2014; Bendell et al., 2015b; Salazar et al., 2015; Seront et al., 2016
BGT-226 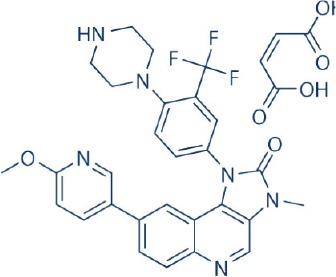	Novartis	Phase I, II	Leukemia, lymphoma, multiple myeloma, breast, head and neck, hepatocellular, pancreatic cancer	Benjamin et al., 2011; Wander et al., 2011; Zhang et al., 2011; Markman et al., 2012
GDC-0980 (Apitolisib) 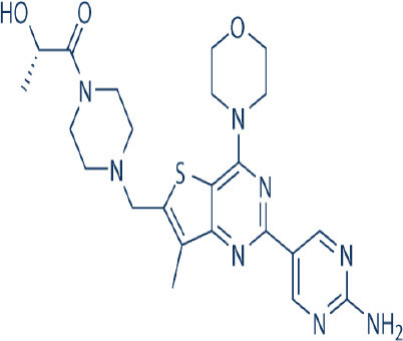	Genentech	Phase I, II	Non-Hodgkin's lymphoma, breast, endometrial, hepatocellular, lung, pancreatic, prostate, renal cancer, malignant pleural mesothelioma	Dolly et al., 2010; Benjamin et al., 2011; Wagner et al., 2011; Wallin et al., 2011; Wander et al., 2011; Zhang et al., 2011; Makker et al., 2014; Powles et al., 2014
GSK-2126458 (Omipalisib) 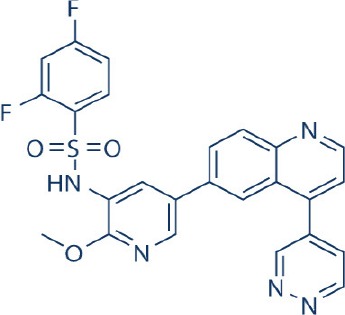	GlaxoSmithKline	Phase I	Lymphoma, breast nasopharyngeal, prostate cancer	Knight et al., 2010; Benjamin et al., 2011; Wander et al., 2011; Zhang et al., 2011; Liu et al., 2015; Munster et al., 2016
PF-04691502 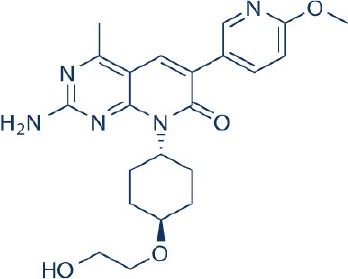	Wyeth/Pfizer	Phase I, II	Leukemia, non-Hodgkin's lymphoma, bladder, breast, colon, endometrial, head and neck, hepatocellular, nasopharyngeal, ovarian cancer	Benjamin et al., 2011; Wander et al., 2011; Yuan et al., 2011; Wang F.-Z. et al., 2013; Britten et al., 2014; Chen et al., 2016
PI-103 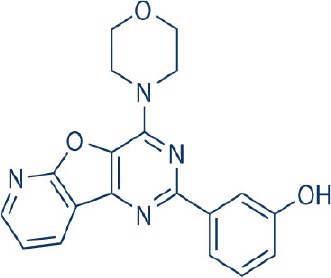	Merck	Preclinical	Leukemia, hepatocellular, lung cancer, astrocytoma, chordomas, glioblastomia, melanoma, neuroblastoma, peripheral nerve sheath tumor	Park et al., 2008
PKI-587 (PF-05212384, Gedatolisib) 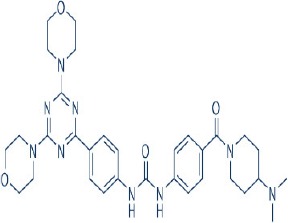	Wyeth/Pfizer	Phase I, II	Leukemia, breast, colon, head and neck, hepatocellular, lung, nasopharyngeal cancer, glioma	Benjamin et al., 2011; Mallon et al., 2011; Wander et al., 2011; Liu et al., 2015
XL-765 (SAR245409, Voxtalisib) 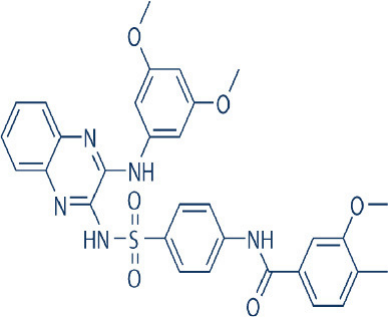	Exelixis Inc.	Phase I, II	Leukemia, lymphoma, breast, pancreatic, prostate cancer, gliomas, neuroblastoma, peripheral nerve sheath tumor, pituitary adenoma	Benjamin et al., 2011; Wander et al., 2011; Zhang et al., 2011; Papadopoulos et al., 2014; Yu et al., 2014; Papadopoulos et al., 2015; Gravina et al., 2016; Thijssen et al., 2016
VS-5584 (SB-2343) 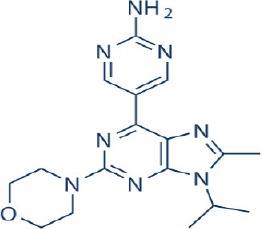	Verastem Inc.	Phase I, II	Leukemia, lymphoma, breast, colon, gastric, ovarian, prostate cancer, melanoma, mesothelioma	Hart et al., 2013
WJD-008 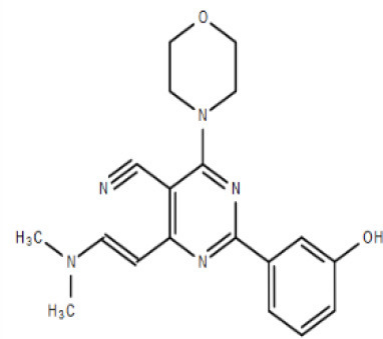	–	Preclinical	Breast, colon, lung, prostate cancer, glioblastoma	Li et al., 2010; Zhou and Huang, 2012
SF-1126 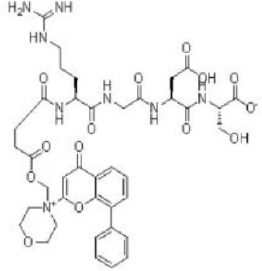	Semafore	Phase I, II	Multiple myeloma, breast, prostate, neck, renal cancer, glioblastoma, neuroblastoma	Benjamin et al., 2011; Zhang et al., 2011; Mahadevan et al., 2012

Among the members of this class, many demonstrate effective anticancer actions with half maximal inhibitory concentration values within the sub-micromolar to low nanomolar range (Zhang et al., 2011). They showed a wide spectrum of activity against many tumor cell lines *in vitro* including those harboring PIK3CA mutations, PTEN and LKB deletion (Maira et al., 2008; Serra et al., 2008; Guillard et al., 2009; Hong et al., 2014; Yu et al., 2014). As with selective mTORC1/mTORC2 inhibitors, dual PI3K/mTOR inhibitors were found to be more efficient at inhibiting the activation of downstream effectors of mTOR including AKT, S6K1, and 4EBP1 compared with rapalogs (Serra et al., 2008; Yuan et al., 2011; Yu et al., 2014). In line with the rationale for their design, downregulation of PI3K-mTORC2 mediated AKT phosphorylation as well as avoidance of PI3K reactivation was also observed after treatment with several PI3K-mTOR dual inhibitors (Maira et al., 2008; Mallon et al., 2011). These effects were in turn translated to G1 cell cycle arrest (Yuan et al., 2011; Wang F.-Z. et al., 2013; Kashiyama et al., 2014; Yu et al., 2014; Chen et al., 2016) or/and apoptosis induction (Park et al., 2008; Brachmann et al., 2009; McMillin et al., 2009; Cho et al., 2010; Mallon et al., 2010; Wallin et al., 2011; Wang F.-Z. et al., 2013; Kashiyama et al., 2014). Some of them also showed the ability to trigger autophagy (Liu et al., 2009; Hong et al., 2014) while others have antiangiogenic properties (Liu et al., 2009; Marone et al., 2009; Wang F.-Z. et al., 2013; Yu et al., 2014). The antitumor effects of these compounds were also observed in numerous xenograft models such as breast cancer (Schnell et al., 2008; Serra et al., 2008; Brachmann et al., 2009; Cao et al., 2009; Marone et al., 2009; Knight et al., 2010; Mallon et al., 2011; Wallin et al., 2011; Yu et al., 2014), pancreatic cancer (Cao et al., 2009), melanoma (Marone et al., 2009), multiple myeloma (McMillin et al., 2009), glioma (Liu et al., 2009; Mallon et al., 2011), renal cell carcinoma (Cho et al., 2010), nasopharyngeal cancer (Liu et al., 2015), colorectal cancer (Mallon et al., 2011), lung cancer (Mallon et al., 2011; Wallin et al., 2011; Yuan et al., 2011), prostate cancer (Serra et al., 2008; Wallin et al., 2011; Gravina et al., 2016), hepatocellular carcinoma (Wang F.-Z. et al., 2013), ovarian cancer (Yuan et al., 2011; Kashiyama et al., 2014), AML (Park et al., 2008), chronic lymphocytic leukemia (Thijssen et al., 2016), and non-Hodgkin's lymphoma (Chen et al., 2016).

The encouraging preclinical effects of dual PI3K-mTOR inhibitors have led to the initiation of numerous clinical studies on many members of this group with good pharmacokinetic properties (Dolly et al., 2010; Wagner et al., 2011; Mahadevan et al., 2012; Markman et al., 2012; Britten et al., 2014; Papadopoulos et al., 2014, 2015; Yokota et al., 2014; Bendell et al., 2015b; Munster et al., 2016). Results of phase I studies of BEZ235, PF04691502, GDC-0980, SF1126, GSK2126458, and XL765 as a single agent in patients with advanced tumors or refractory lymphoma have been reported (Papadopoulos et al., 2014, 2015). In general, PI3K blockage and reduction in phosphorylated AKT level were observed in only a portion of tumor biopsies analyzed (Dolly et al., 2010; Wagner et al., 2011; Mahadevan et al., 2012; Markman et al., 2012; Britten et al., 2014; Papadopoulos et al., 2014; Yokota et al., 2014; Bendell et al., 2015b; Papadopoulos et al., 2015; Munster et al., 2016). Objective response was not observed in solid tumor patients treated by these agents while only a subset of these patients exhibited stable disease (Dolly et al., 2010; Wagner et al., 2011; Mahadevan et al., 2012; Markman et al., 2012; Britten et al., 2014; Papadopoulos et al., 2014; Yokota et al., 2014; Bendell et al., 2015b; Papadopoulos et al., 2015; Munster et al., 2016). In patients with solid tumor treated with GSK2126458, antitumor activity of this dual PI3K-mTOR inhibitor was independent of patient's PIK3CA mutational status (Munster et al., 2016). On the other hand, in 12 lymphoma patients treated with XL765, 1 had complete response while 2 other patients achieved partial response following treatment (Papadopoulos et al., 2014). In these studies, common adverse drug reactions observed include fatigue, hyperglycemia, rash, and poor appetite (Dolly et al., 2010; Wagner et al., 2011; Mahadevan et al., 2012; Markman et al., 2012; Britten et al., 2014; Papadopoulos et al., 2014; Yokota et al., 2014; Bendell et al., 2015b; Papadopoulos et al., 2015; Munster et al., 2016). In patient treated with XL765, an increase in transaminases was the most common high grade toxicity (Papadopoulos et al., 2014, 2015). The outcome of a phase II study of endometrial cancer patients receiving 40 mg of oral GDC-0980 daily was also available (Makker et al., 2014). In this study, 20% of patient experienced progression free survival at 6 months with objective response seen only in 9% of patients (Makker et al., 2014). Among the tumor samples tested, 52% of samples had at least one alteration in PIK3CA, PTEN, or AKT. Dose reduction was however seen in 39% of enrolled patients with many experiencing grade 3 or higher hyperglycaemia, rash, diarrhea, or fatigue (Makker et al., 2014). Here, diabetic patients were observed to have poorer tolerability to GDC-0980 compared to non-diabetics (Makker et al., 2014). In another phase II study of GDC-0980 vs. evorolimus in metastatic renal cell carcinoma, median PFS in was shorter in GDC-0980 patients compared with everolimus (3.7 vs. 6.1 month; Hazard Ratio = 2.04, Confidence interval: 1.18–3.54; *p* < 0.01), indicating the lack of benefit of GDC-0980 over everolimus in this group of patient (Powles et al., 2014). In addition, a high incidence of adverse events associated with the use of GDC-0980 was observed (Powles et al., 2014). Besides GDC-0980, results of two phase II studies of BEZ235 monotherapy in advanced pancreatic neuroendocrine tumors and transitional cell carcinoma have also been reported. In patients with pancreatic neuroendocrine tumors, BEZ235 as with comparator everolimus showed limited clinical activity against this cancer. Moreover, in this trial, BEZ235 showed poorer tolerability compared with everolimus (Salazar et al., 2015). In a similar manner, BEZ235 only showed modest clinical effects as a monotherapy in patients with transitional cell carcinoma. In this study, an unfavorable toxicity profile with 40% of patient experiencing high grade adverse events was observed (Seront et al., 2016).

Thus far, the mTOR modulating actions of these inhibitors as monotherapy in cancer have only achieved modest effects as most agents only resulted in stable disease rather than objective response which is typified by tumor regression in most cancers (Carew et al., 2011). This has been attributed to the cytostatic effect of these compounds as single agents and activation of compensatory signaling pathways (Keck et al., 2012). Therefore, it has been suggested that combined use of these compounds with other anticancer agents will afford better anticancer response. Numerous anticancer agents including conventional cytotoxic drugs, targeted therapy, and radiation have been tested with various mTOR inhibitors in preclinical models (Geoerger et al., 2001; Mondesire et al., 2004; Prevo et al., 2008; Baumann et al., 2009; Westhoff et al., 2009; Manara et al., 2010; Fokas et al., 2012). Several drug combinations have resulted in additive or synergistic effects compared to single agent treatment in these preclinical studies (Geoerger et al., 2001; Mondesire et al., 2004; Baumann et al., 2009; Westhoff et al., 2009; Manara et al., 2010). For example, rapamycin has been shown to potentiate the cytotoxicity of paclitaxel, carboplatin, and vinorelbine in breast cancer cell lines (Mondesire et al., 2004). Combined use of PI-103 with doxorubin or etoposide or radiation resulted in increased apoptosis compared to single agent alone (Fokas et al., 2012). Likewise, concurrent use of BEZ235 with melphalan, doxorubicin and bortezomib caused synergistic cytotoxicity in Ewing sarcoma cells (Manara et al., 2010).

As a result of these encouraging data, various clinical studies have since been performed or are currently underway to evaluate the benefit of mTOR inhibition in addition to targeting other oncogenic processes (Clinical trial identifiers for these studies are available at ClinicalTrials.gov). To date, most data is available for combination therapy based on rapalogs. Outcomes of these trials again showed high variability (Baselga et al., 2012; Wolff et al., 2013; André et al., 2014; Hurvitz et al., 2015). mTOR modulation with addition of everolimus to carboplatin resulted in effective disease control among some treated patients with triple negative breast cancer (Singh et al., 2014). Similarly, concurrent use of everolimus with exemestane or trastuzumab plus vinorelbine in breast cancer patients, who are resistant to aromatase inhibitors or trastuzumab respectively, have led to significant improvement in PFS, indicating that reducing mTOR activity is useful against chemoresistance in breast cancer (Baselga et al., 2012; André et al., 2014). On the hand, addition of everolimus to carboplatin in patients with metastatic prostate cancer demonstrated minimal efficacy (Vaishampayan et al., 2015). Combined use of rapalogs with epidermal growth factor receptor inhibitors in glioblastoma or lung cancer have also resulted in no survival benefit or greater toxicities respectively (Kreisl et al., 2009; Price et al., 2010; Reardon et al., 2010). Details of more studies on combinatorial use of rapalogs can be found in several recent reviews (Lauring et al., 2013; Yardley, 2013; Ortolani et al., 2015).

At present, data on combined therapy of selective mTORC1/mTORC2 or dual PI3K/mTOR inhibitors with other anticancer agents are limited as many clinical trials are currently ongoing (Clinical trial identifiers for these studies are available at ClinicalTrials.gov). Results from an early phase Ib study of GDC-0980 in combination with carboplatin, paclitaxel with or without bevacizumab or GDC-0980 with cisplatin and pemetrexed in patients with advanced cancer or non-small cell lung cancer (NSCLC) is encouraging with preliminary activity of these combinations observed as in NSCLC (Calvo et al., 2014).

Overall, current evidence indicates that modulation of mTOR activity with mTOR inhibitors in selected tumors is useful in preventing their progression and overcoming their chemoresistance to other anticancer agents.

## mTOR inhibitors for modulating mTOR activity to combat cancer stem cells

The notion that therapeutic targeting of mTOR may be useful in eradicating CSCs originates from the prevailing evidence that mTOR signaling is activated in CSCs while its tight regulation is required for maintenance of normal stem cell pool (Yilmaz et al., 2006). Initial studies using rapamycin to verify the involvement of mTOR signaling for tumorigenic formation of CSCs that were discussed previously provided a convincing basis that targeting the mTOR pathway using inhibitors may eradicate CSCs which can in turn augment overall cancer treatment. Several other studies have further illustrated the inhibitory effects of rapamycin on CSCs (Chang et al., 2013; Matsubara et al., 2013; Wang Y. et al., 2013; Cai et al., 2014). In a study by Cheng et al., rapamycin treatment reduced the percentage of aldehyde dehydrogenase positive (ALDH+) breast CSCs and inhibited mammosphere formation capacity of human breast cancer xenografts (Chang et al., 2013). Similar results were also obtained in pancreatic and colon cancers. Matsubara and colleagues found that rapamycin reduced the viability and stemness characteristics of CD133+ pancreatic CSCs (Matsubara et al., 2013). For the latter, Wang and co-workers showed that rapamycin inhibited stemness, epithelial-mesenchymal transition, and invasive of colorectal CSCs induced by FBXW7 deficiency (Chang et al., 2013). In addition, Cai et al. reported the ability of rapamycin to decrease ALDH activity and sphere formation in CSCs derived from one out of the two colorectal cancer cell lines tested (Cai et al., 2014).

More importantly, available evidence suggests that the newly developed second generation mTOR inhibitors have stronger preferential inhibitory effect on CSCs compared to normal stem cells than rapamycin or rapalogs (Cai et al., 2014; Kolev et al., 2015). In colon cancer, PP242, a selective mTORC1/mTORC2 inhibitor, reduced ALDH activity of CSCs derived from both colorectal cancer cell lines investigated (Cai et al., 2014). PP242 but not rapamycin also suppressed the enrichment of ALDH+ CSCs induced by 5-fluorouracil or oxaliplatin treatment (Cai et al., 2014). In a MCF-7 breast cancer xenograft, VS-5584, a dual PI3K-mTOR inhibitor, but not everolimus reduced the proportion of CSCs following drug administration (Kolev et al., 2015). VS-5584 further exhibited preferential targeting of CSCs and delayed tumor regrowth after chemotherapy in primary ovarian and small cell lung cancer xenografts (Kolev et al., 2015). Other *in vitro* and *in vivo* studies have since identified more second generation mTOR inhibitors with CSCs inhibitory properties (Dubrovska et al., 2009; Fang et al., 2013). For example, BEZ235 was capable of preventing the growth of prostate CSCs (Dubrovska et al., 2009) while PF-04691502 reduced the proliferation of PIK3CA (H1047R) mutant colorectal CSCs *in vitro* and *in vivo* (Fang et al., 2013).

Although monotherapy of second generation mTOR inhibitors showed promising activity against CSCs in preclinical studies, combinatorial use of rapamycin or these inhibitors with other signal transduction inhibitors or conventional chemotherapeutics/radiotherapy has been promulgated. This arises from the idea that stemness of CSCs can be caused by numerous pathways with mTOR signaling being only one of them (Fitzgerald et al., 2015). Furthermore, significant crosstalk can occur between these signaling systems (Brechbiel et al., 2014), hence simultaneously targeting of numerous nodes important in CSCs survival can more effectively eradicate these cells (Fitzgerald et al., 2015). From another perspective, it is generally believed that conventional anticancer drugs target the rapidly proliferating bulk tumor cells while sparing CSCs (Sehl et al., 2009). The surviving CSCs can in turn cause cancer relapse (Sehl et al., 2009). Combined use of these treatment modalities with mTOR inhibitors that are more effective at targeting CSCs thereby renders more effective tumor destruction, better disease control and overcome the CSCs-mediated tumor resistance associated with the use of traditional agents (Sehl et al., 2009). These hypotheses have already been validated experimentally by several groups of investigators. In pancreatic CSCs, Mueller and co-workers showed that concurrent targeting of sonic Hedgehog (HH) signaling, another pathway activated in pancreatic CSCs, using cyclopamine, a HH inhibitor, with rapamycin successfully eliminated the pancreatic CSC pool while either agent alone was ineffective (Mueller et al., 2009). This was in turn translated into significantly improved survival of mice bearing these patient-derived CSC xenografts (Mueller et al., 2009). In a separate study, combined use of a recepteur d'origine nantais (RON) receptor tyrosine kinase inhibitor, BMS-777607, with AZD8055 or PP242 resulted in synergistic kill of pancreatic CSCs and chemoresistant pancreatic cancer cells (Zeng et al., 2014). In liver cancer stem cell (LCSC) lines, simultaneous treatment of sorafenib with PKI-587 resulted in a 47% and 19% increase in inhibition of LCSC cell proliferation compared with sorafenib or PKI-587 lone treatment respectively (Gedaly et al., 2013). For breast cancer, concurrent use of everolimus with trastuzumab resulted in greater reduction in the *in vitro* tumorigenicity as well as *in vivo* growth of patient derived and BT474 CSCs than either agent alone (Zhu et al., 2012). Addition of rapamycin, everolimus, or PF-04691502 to tamoxifen therapy also reduced mammosphere formation of patient derived CSCs through ameliorating tamoxifen induced mTOR activation in these CSCs (Karthik et al., 2015). Likewise, in glioblastoma, pretreatment with AZD2014 followed by radiation or combined therapy of BEZ235 with radiation increased the radiosensitivity of glioblastoma CSCs *in vitro* (Wang W.-J. et al., 2013; Kahn et al., 2014). In the former study, AZD2014 retained its ability to enhance the radiosensitivity of orthotopic glioblastoma CSC xenografts due to its ability to penetrate the blood brain barrier (Kahn et al., 2014). In both studies, enhancement of CSCs kill was due to inhibition of DNA double strand break repair (Wang W.-J. et al., 2013; Kahn et al., 2014). BEZ235 enhancement of radiosensitivity of CSC was also accompanied by cell cycle arrest, autophagy and apoptosis induction (Wang W.-J. et al., 2013). As a whole, targeting the mTOR axis represents a potentially useful strategy for eradication of CSCs and reduction of tumor burden in selected type of cancer.

## Perspectives and conclusion

mTOR is a druggable target implicated in the development of IR and cancer. Careful manipulation of the activities of mTORC1 and mTORC2 complexes may aid in the management of these pathological conditions. For combating IR, care must be taken to avoid mTORC2 inhibition and prolonged reduction of mTORC1 activity. Low dose, intermittent rapamycin use to carefully manage overactivation of mTORC1 is a potential strategy to sensitize cells to the action of insulin. Currently, no human study has been done in this area. Clinical trials should be performed in the future to elucidate a suitable dosing schedule for low dose rapamycin therapy. Additionally, combinatorial use of low dose rapamycin with other agents capable of ameliorating IR should be explored in clinical studies. Two such combinations include concurrent use of low dose rapamycin with either metformin or resveratrol. For the former, the ability of metformin to inhibit hepatic gluconeogenesis may be beneficial for rapamycin therapy since rapamycin use has been known to induce gluconeogenesis. Such mechanism based rationale drug combination may make the use of low dose rapamycin safer. For the latter, combined use of resveratrol with rapamycin has already been shown in murine model to potentiate the action of rapamycin at preventing hyperinsulinemia (Leontieva et al., 2013). This effect has been shown to be mediated by the inhibition of S6K1 and other unknown mechanisms of resveratrol (Chen and Huang, 2013; Leontieva et al., 2013).

In cancer treatment, efficacy of mTOR inhibitors in clinical practice is limited to a subset of patient. This has been attributed to a lack of clinically useful predictive markers to guide the use of these agents in patients that are most likely going to benefit from them. Although several preclinical studies and analysis of archival tumor samples from patients who have participated in various trials have identified candidate genetic and non-genetic response markers, these markers may be tumor specific (Weigelt and Downward, 2012; Sun, 2013a; Paplomata and O'Regan, 2014). Therefore, a more global genetic and non-genetic analysis is required for different tumor types to identify the different markers that may influence their outcome to mTOR inhibitor treatment. As it has been suggested that different clones of CSCs may have contributed to the heterogeneity of cells within a tumor mass, biomarkers identification should be performed on multiple biopsy samples so as to derive the most useful panel of predictive markers.

Apart from predictive biomarkers identification, resistance to mTOR inhibitors have been increasingly reported (Czarnecka et al., 2014). It is therefore important to also identify markers of resistance so as to better understand the compensatory pathways that are activated following the use of different classes of mTOR inhibitors. Through this, more rationale combinatorial therapy can be designed together with these inhibitors so as to optimize the use of these agents.

In conclusion, mTOR signaling plays an important role in IR and tumorigenesis. Its careful modulation is crucial to the management of these pathological conditions. Studies to identify rational rapamycin containing regimens may provide an alternative strategy for ameliorating IR. In cancer treatment, data from ongoing trials of second generation mTOR inhibitors may offer opportunities for treating cancers with aberrant mTOR activities. Improvement to mTOR targeted therapy can be further achieved through careful stratification of patients based on predictive biomarkers identified and use of mechanistically synergistic combinations.

## Author contributions

PO, LW, and GS conceived and designed the manuscript. All authors contributed to the writing.

### Conflict of interest statement

The authors declare that the research was conducted in the absence of any commercial or financial relationships that could be construed as a potential conflict of interest.
